# Prophylactic Negative-Pressure Wound Therapy After Laparotomy: Ongoing Discussion Following High-Quality Systematic Review

**DOI:** 10.1007/s00268-023-06948-z

**Published:** 2023-02-25

**Authors:** Andreas Älgå, Jonas Malmstedt

**Affiliations:** 1 Department of Clinical Science and Education, Södersjukhuset, Karolinska Institutet, Sjukhusbacken 10, 118 83 Stockholm, Sweden; 2 Department of Global Public Health, Karolinska Institutet, 171 77 Stockholm, Sweden

## Commentary

Since the introduction of negative-pressure wound therapy in the early 1990s, commercial companies and independent researchers have struggled to produce high-quality evidence of the technique’s effectiveness [1]. The further development *prophylactic* negative-pressure wound therapy (pNPWT) is increasingly being promoted as a means to prevent surgical site infection (SSI) of closed surgical incisions [1]. However, due to the low quality of evidence, the World Health Organization recommends limiting the use of pNPWT to high-risk conditions, such as poor tissue perfusion, decreased blood flow, dead space, or intraoperative contamination [2]. The effectiveness and cost-effectiveness of pNPWT to prevent SSI after laparotomy have been assessed in observational studies and randomized controlled trials (RCTs) with contradictory results [1, 3].

The systematic review by Jeremy Meyer et al. provides a thorough meta-analysis of the available data from RCTs evaluating pNPWT after laparotomy [3]. We commend the authors for the sound methodology and the transparency regarding bias and quality issues among the included studies. This information is highly relevant as the conclusions that can be drawn from systematic reviews and meta-analyses are always determined by the quality of the included studies – an RCT design does not guarantee high-quality evidence. All but one of the eleven included studies were considered at high risk of bias [3]. A majority of the RCTs did not use blinded outcome assessors, and 8/11 (73%) of the trials were supported by the industry. The reader should also bear in mind the small-study effect and publication bias, as alluded to by the authors. In addition, the reviewed RCTs used a wide definition of SSI and trial outcomes included superficial SSIs that might not be clinically important. If the effect of pNPWT is evaluated based on the reduction of superficial incisional SSIs with uncertain clinical significance, the cost-effectiveness of pNPWT has to be assessed accordingly. We suggest that, rather than SSI, wound dehiscence might be more clinically relevant to use as primary outcome measure. However, a recent Cochrane review concluded that there is probably no difference in wound dehiscence among people treated with pNPWT compared with standard treatment (moderate‐certainty evidence) [4].

In a wider perspective, one could ask to whom the results from an RCT apply. In the systematic review by Meyer et al., there is a considerable heterogeneity in the studied populations, e.g., type of surgery, acute vs. elective surgery, clean vs. contaminated wounds, and patient-related risk factors for SSI, such as obesity, diabetes mellitus, and American Society of Anesthesiologists (ASA) score. Pooling data from studies with such variation in inclusion and exclusion criteria will limit the generalizability of the findings. Until the effect of pNPWT in the prevention of SSI after laparotomy is established, the cost-effectiveness of the technique cannot be fully assessed. In low- and middle-income countries, evidence-based low-cost interventions to prevent SSI, such as sterile glove and instrument change at the time of abdominal wound closure, preoperative skin preparation with chlorhexidine alcohol, and timely administration of antibiotic prophylaxis, should be implemented before the introduction of costly techniques without evidence of cost-effectiveness [5, 6].

We conclude that the value of pNPWT after laparotomy remains uncertain. Before pNPWT can be considered in routine care after laparotomy, a large, high-quality RCT is needed. We propose the design of a multinational, non-industry-funded trial with clinically meaningful outcome measures utilizing blinded and independent assessors to further establish in which settings and for which patient groups pNPWT might be effective and cost-effective.

## Abbreviations

ASA: American Society of Anesthesiologists
pNPWT: Prophylactic negative-pressure wound therapy
RCT: Randomized controlled trial
SSI: Surgical site infection
